# Targeting Endothelial Connexin37 Reduces Angiogenesis and Decreases Tumor Growth

**DOI:** 10.3390/ijms23062930

**Published:** 2022-03-08

**Authors:** Karthik Sathiyanadan, Florian Alonso, Sonia Domingos-Pereira, Tania Santoro, Lauriane Hamard, Valérie Cesson, Paolo Meda, Denise Nardelli-Haefliger, Jacques-Antoine Haefliger

**Affiliations:** 1 Department of Urology, Lausanne University Hospital, 1011 Lausanne, Switzerland; karthik.sathiyanadan@gmail.com (K.S.); sonia.domingos-pereira@chuv.ch (S.D.-P.); valerie.cesson@chuv.ch (V.C.); dnardell@hospvd.ch (D.N.-H.); 2 Laboratory for the Bioengineering of Tissues (BioTis-INSERM U1026), Université de Bordeaux, 33607 Bordeaux, France; f.alonso@iecb.u-bordeaux.fr; 3 Department of Medicine, Lausanne University Hospital, 1011 Lausanne, Switzerland; tania.santoro@chuv.ch (T.S.); lauriane.hamard@chuv.ch (L.H.); 4 Department of Cell Physiology and Metabolism, Medical Center, University of Geneva, 1206 Geneva, Switzerland; paolo.meda@unige.ch

**Keywords:** cell-cell communication, connexins, angiogenesis, tumors, transgenic mice

## Abstract

Connexin37 (Cx37) and Cx40 form intercellular channels between endothelial cells (EC), which contribute to the regulation of the functions of vessels. We previously documented the participation of both Cx in developmental angiogenesis and have further shown that loss of Cx40 decreases the growth of different tumors. Here, we report that loss of Cx37 reduces (1) the in vitro proliferation of primary human EC; (2) the vascularization of subcutaneously implanted matrigel plugs in Cx37−/− mice or in WT using matrigel plugs supplemented with a peptide targeting Cx37 channels; (3) tumor angiogenesis; and (4) the growth of TC-1 and B16 tumors, resulting in a longer mice survival. We further document that Cx37 and Cx40 function in a collaborative manner to promote tumor growth, inasmuch as the injection of a peptide targeting Cx40 into Cx37−/− mice decreased the growth of TC-1 tumors to a larger extent than after loss of Cx37. This loss did not alter vessel perfusion, mural cells coverage and tumor hypoxia compared to tumors grown in WT mice. The data show that Cx37 is relevant for the control of EC proliferation and growth in different tumor models, suggesting that it may be a target, alone or in combination with Cx40, in the development of anti-tumoral treatments.

## 1. Introduction

Angiogenesis, i.e., the formation of new blood vessels, is critical for providing the oxygen and nutrients which are necessary for tumor growth [1,2,3]. It requires a fine-tuned coordination between the proliferation, migration and differentiation of both endothelial (EC) and mural cells [4,5]. When exposed to various angiogenic factors, notably vascular endothelial growth factor (VEGF) [6,7], quiescent vessels sprout due to the local proliferation and migration of EC [5,8,9]. The further vascular maturation of the newly formed endothelial tubes is carried out by various factors secreted by EC, including PDGF isoforms, which act as chemo-attractants for mural cell precursors [10,11]. During tumor angiogenesis, the overexpression of pro-angiogenic factors results in the formation of irregular and tortuous vessels, which feature a discontinuous pericyte coverage, a high permeability [1,12] and a fenestrated basement membrane [13]. These features compromise the barrier integrity of the tumor vasculature and its perfusion, resulting in a sustained hypoxia, which may contribute to tumor maintenance and resistance to chemotherapy [14,15].

Connexins (Cx) form channels for the electrical and metabolic signaling, which coordinates the functions of individual cells [16,17], notably within the wall of blood vessels [18,19,20]. Between EC, this signaling is mediated by Cx40 and Cx37 [21,22,23,24,25]. The combined deletion of these two Cx causes lethal, perinatal hemorrhages, suggesting that both proteins are required for the normal development of the microvasculature [26,27]. We previously demonstrated that Cx40 contributes to regulating the structure and function of vessels during tumoral angiogenesis [28], since the loss of this Cx reduces angiogenesis and tumor growth in mice, while increasing vessel perfusion, mural cell coverage and animal survival. We further documented that loss of EC Cx40 also delays the physiological angiogenesis in the developing mouse retina [29].

Given that the loss of Cx37 was also found to alter the development and maturation of retinal vasculature [30,31] and that mutations in the *GJA4* gene, which codes for Cx37 in humans, result in vascular malformations [32,33], we hypothesized that Cx37 could also participate in the angiogenesis of developing tumors. To test this possibility, we compared WT and Cx37−/− mice [34], which show control levels of Cx40 in EC [21,35]. Compared to WT littermates, Cx37−/− mice feature an increased bone mass [36,37] and body weight [38]. Moreover, Cx37−/− female mice are infertile due to defects in follicular growth, luteinization and oocyte maturation [34].

Here, we report that, in vivo, loss of Cx37 reduces the vascularization in matrigel plugs as well as in different tumor models, prolonging the survival of the tumor-bearing mice. The specific contribution of Cx37 to angiogenesis was further confirmed by using a Cx37 inhibitory peptide, which resulted in a decreased neovascularization of matrigel plugs [39,40]. In contrast with the observations previously made in Cx40−/− mice [28], we found that loss of Cx37 did not modify the perfusion and mural cells coverage of the newly formed vessels; rather, it reduced the in vitro proliferation of EC. We also observed that the combined targeting of Cx37 and Cx40 signaling, after injection of a Cx40 inhibitory peptide into Cx37−/− mice, resulted in a further reduction of tumor growth, suggesting that the two Cx function in a collaborative manner to control tumoral angiogenesis and growth. The data identify Cx37 as a significant partner in the control of tumoral angiogenesis, which, in addition to Cx40, could be a valuable target for innovative anti-cancer treatments.

## 2. Results

### 2.1. Loss of Cx37 Decreases Angiogenesis

**Figure 1:**
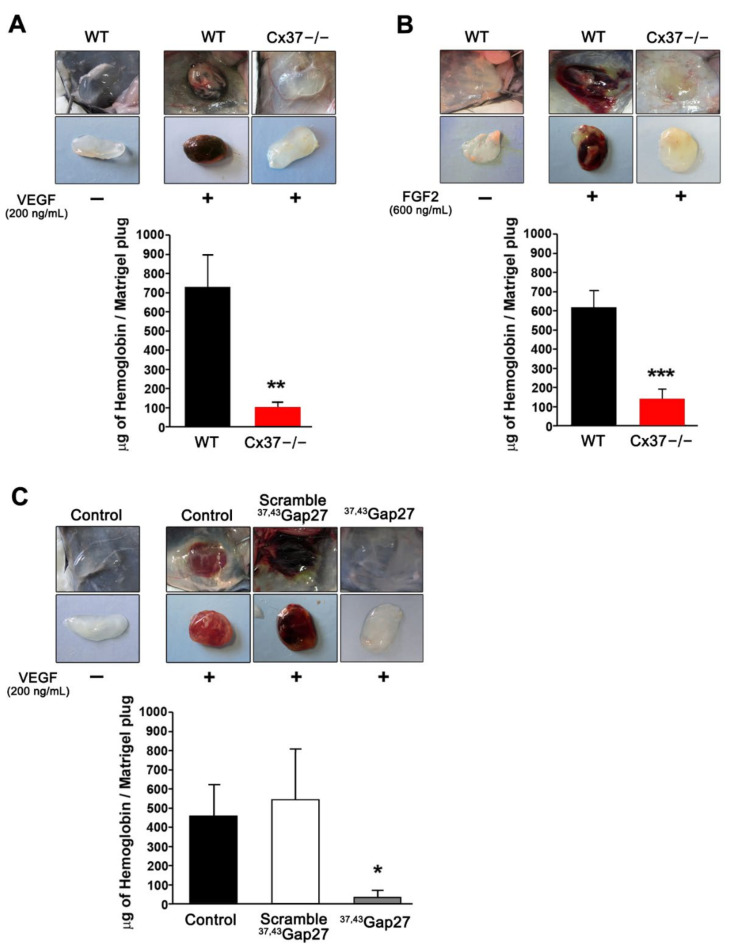
Loss of Cx37 reduces angiogenesis in subcutaneous matrigel plugs. (**A**,**B**) Representative pictures of matrigel plugs without and with VEGF (**A**) or FGF2 (**B**), one week after s.c. injection in WT and Cx37−/− mice. In the absence of vascular growth factors, the plugs retained their original whitish color, due to the absence of vascularization. In the presence of the growth factors, the plugs implanted in WT mice acquired a red-brown color, reflecting their neovascularization, as also judged by a sizable hemoglobin content. Both the color change and the hemoglobin content of the plugs were significantly lower in the plugs implanted in Cx37−/− mice. Data are means + SEM. *n* = 13 WT and 9 Cx37−/− mice. ** *p <* 0.01, *** *p* < 0.001 versus WT mice (Student’s *t*-test). (**C**) Matrigel plugs containing VEGF were supplemented with either the ^37,43^Gap27 peptide or its scrambled version, before implantation in WT mice. The peptide targeting Cx37 markedly reduced the color change and the hemoglobin content of the plugs, which retained the whitish appearance of the controls devoid of VEGF, and which was similar to that observed in Cx37−/− mice. In contrast, plugs loaded with VEGF and the scrambled peptide became red-stained and featured a large increase in hemoglobin content, as observed in WT mice. Data are mean + SEM. *n* = 5–6 mice per group. * *p* < 0.05 versus scramble (one-way ANOVA followed by Tukey’s post-test).

One week after the s.c. implantation in WT mice, matrigel plugs devoid of angiogenic growth factors featured a whitish appearance (Figure 1A,B), suggesting no significant angiogenesis. In contrast, plugs that had been supplemented with either VEGF (Figure 1A) or FGF2 (Figure 1B) featured an intense red-brown staining and a sizable hemoglobin content, indicating extensive angiogenesis.

In contrast, when implanted in Cx37−/− mice, these plugs remained whitish and showed a significant lower hemoglobin content. Parallel experiments revealed that supplementing the plugs with ^37,43^Gap27, a peptide which inhibits Cx37 channels [39,40,41], reduced the angiogenesis in WT mice, an effect which was not observed in plugs supplemented with a scrambled version of this peptide (Figure 1C). The data demonstrate that reducing the levels of Cx37 or interfering with its function reduced angiogenesis in vivo, in response to different angiogenic stimuli.

### 2.2. Silencing Cx37 Reduces EC Proliferation

**Figure 2:**
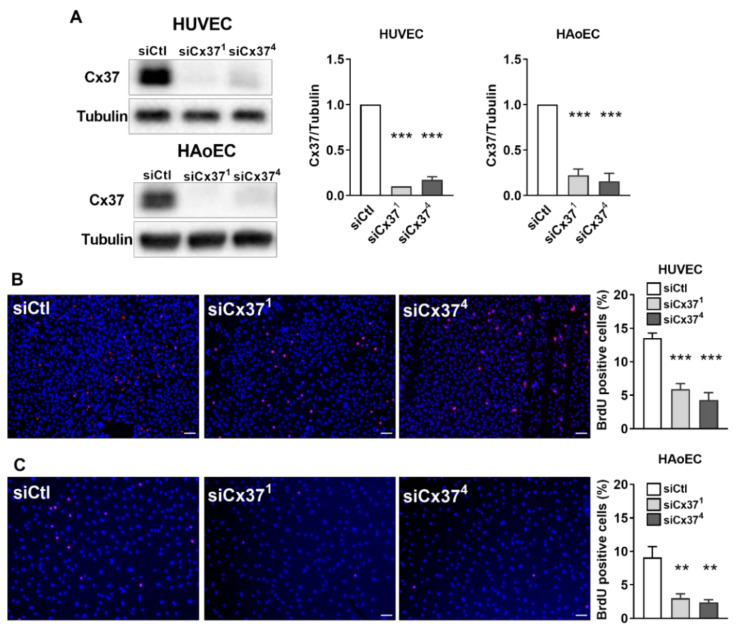
In vitro targeting Cx37 reduces the proliferation of primary EC. (**A**) Western blot of primary human vein (HUVEC) and aortic EC (HAoEC) revealed that Cx37 expression was reduced by 80–90% 48 h after transfection with either siCx37^1^ or siCx37^4^, two siRNAs targeting Cx37. Such a drastic change was not observed when the cells were transfected with a control siRNA (siCtl). Graphs show mean + SEM, *** *p* < 0.001 versus siCtl (one-way ANOVA followed by Tukey’s post-test). (**B**,**C**) As judged by the incorporation of BrdU (red) in the nuclei (stained in blue by DAPI), the proliferation of the HUVEC and HAoEC that had lost most of Cx37 was decreased to about 30% of control levels. Graphs show mean values + SEM, *n* = 8 separate experiments, each performed in duplicate. ** *p* < 0.01, *** *p* < 0.001 vs. siCtl (one-way ANOVA followed by Tukey’s post-test). Scale bars = 50 μm.

To determine whether the loss of Cx37 could interfere with the proliferation of EC, primary HUVEC and HAoEC, which both express Cx37 (Figure 2A), were transfected with either siCx37^1^ or siCx37^4^. Western blot analysis showed that both siRNAs markedly decreased the expression of Cx37 in the two cell types (Figure 2A) and significantly reduced their proliferation, as judged by a decreased BrdU incorporation (Figure 2B,C).

### 2.3. Loss of Cx37 Decreases the Growth and the Vascularization of Tumors

**Figure 3:**
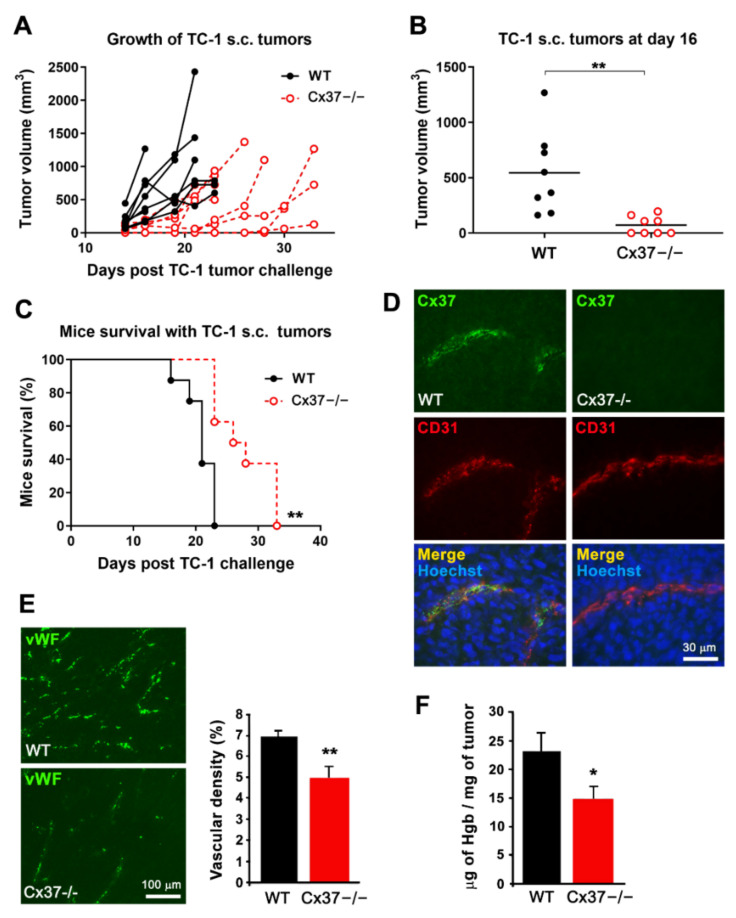
The growth of TC-1 tumors is reduced in Cx37−/− mice. (**A**) After s.c. injection, TC-1 cells generated growing tumors in all WT and Cx37−/− mice. (**B**) Sixteen days after the cell injection, the volume of tumors was significantly lower in Cx37−/− (*n* = 8) than in WT mice (*n* = 8). ** *p* < 0.01 versus WT mice (Student’s *t*-test). The horizontal bars show the mean tumor volume in each group. (**C**) All WT mice carrying the TC-1 tumor were sacrificed within the first 3 weeks of the experiments. In contrast, the cognate Cx37−/− mice survived significantly longer. ** *p* = 0.01 versus WT mice (log-rank Mantel-Cox test). (**D**) Sixteen days after the injection of TC-1 cells, Cx37 was detected via immunostaining in CD31-positive EC of the tumors grown in WT (left) but not Cx37−/− mice (right). Bar, 30 µm. (**E**) Immunostaining of the EC-specific von Willebrand factor (vWF) revealed a lower density of vessels in the tumors grown in Cx37−/− than in WT mice. Data are mean + SEM of 5–7 areas from size-matched tumors that developed in 4 mice per group. ** *p* < 0.01 versus WT mice (Student’s *t*-test). Bar, 100 µm. (**F**) The hemoglobin concentration was also lower in the tumors that developed in Cx37−/− than WT mice. Data are mean + SEM. *n* = 6 mice per group. * *p* < 0.05 vs. WT mice (Student’s *t*-test).

Twelve days after the s.c. implantation of TC-1 cells, sizable tumors were observed in both WT and Cx37−/− mice. After 16 days, these tumors were significantly larger in WT than Cx37−/− mice (Figure 3A,B). Accordingly, all WT mice carrying a tumor were sacrificed after 21 days, whereas Cx37−/− mice survived for significantly longer periods (Figure 3C). Analysis of the tumor vascularization revealed Cx37 only in EC of WT mice vessels (Supplementary Figure S1), which harbored a similar, normal structure of their walls in the presence and absence of Cx37 (Figure 3D). A significantly lower vascular density (Figure 3E) and hemoglobin content (Figure 3F) were observed in the tumors grown in Cx37−/− than in WT mice. Collectively, the data show that lack of Cx37 reduces in vivo the growth and angiogenesis of TC-1 tumors, thus extending the survival of the tumor-bearing mice.

### 2.4. Loss of Cx37 Does Not Alter the Perfusion and the Maturation of Vessels in theTC-1 Tumors

**Figure 4:**
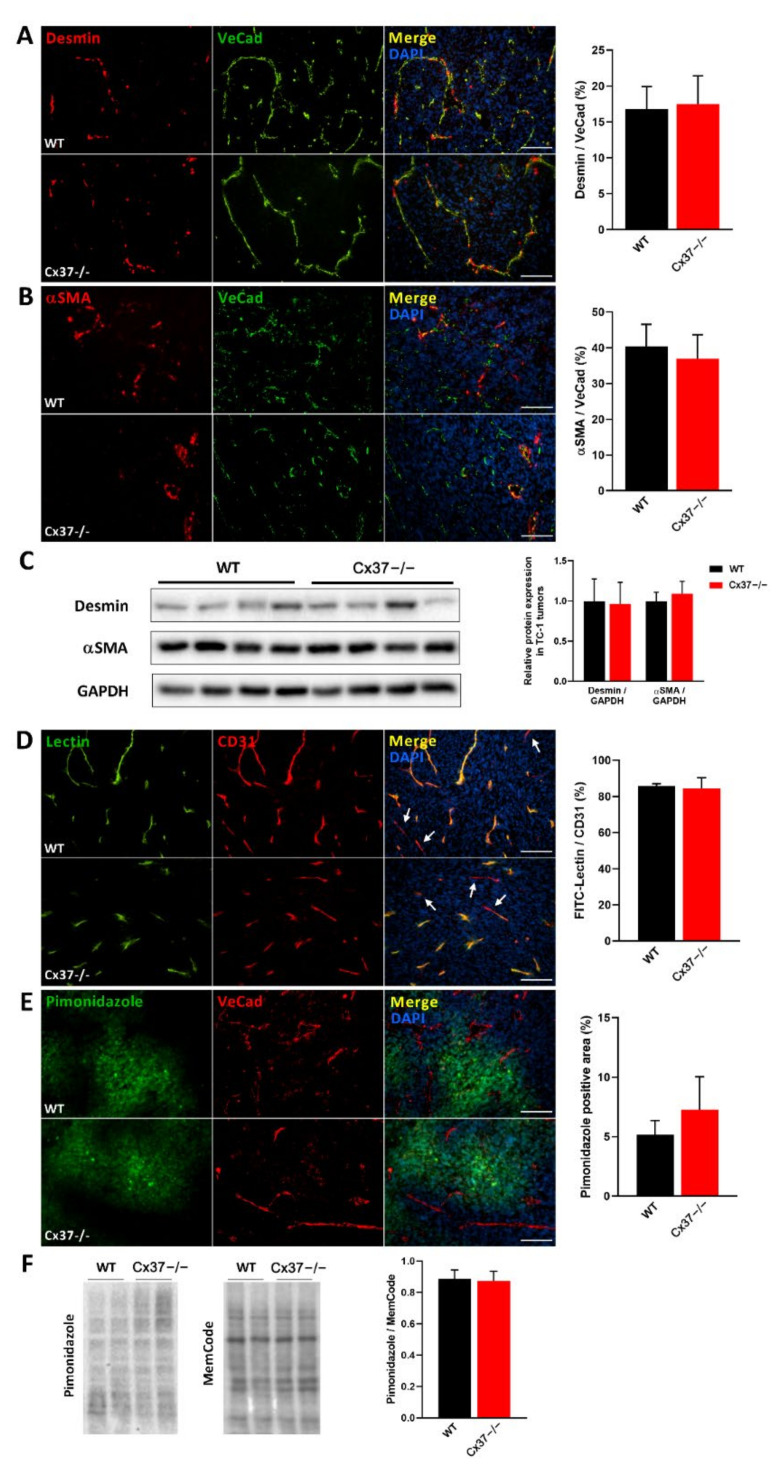
Loss of Cx37 does not alter the maturation of vessels in TC-1 tumors. (**A**,**B**) Left panels: immunostaining of desmin (**A**) and alpha-smooth muscle actin (αSMA; (**B**)) shows the mural cells which surrounded VeCad-positive EC in the newly formed vessels of TC-1 tumors. Right panels: The right bar graphs show that the coverage of vessels by mural cells was similar in the tumors grown in Cx37−/− and WT mice. Data are mean + SEM of 8–15 fields, from at least 5 mice per group. Bars = 100 μm. (**C**) Western blots confirmed that the tumors grown in Cx37−/− and WT mice featured similar levels of desmin and αSMA. Data are mean + SEM. *n* = 7 WT and 8 Cx37−/− mice (Student’s *t*-test). (**D**) Infusion of FITC-labeled tomato lectin (green) stained most CD31-positive vessels (red) of TC-1 tumors in both Cx37−/− and WT mice (white arrows indicate some of the few vessels that were not perfused by the lectin). Quantitative analysis showed that a similar proportion of tumor vessels was perfused in the two types of animals. Data are mean + SEM of 6–8 fields from 4 mice per group (Student’s *t*-test). (**E**) Immunostaining of pimonidazole (green) revealed hypoxic areas in tumor regions containing VeCad-positive vessels (red). Quantitative analysis revealed that the percentage of these hypoxic areas was comparable in the tumors grown in Cx37−/− and WT mice. Data are mean + SEM of 8–15 fields from 7 mice per group (Student’s *t*-test). Bars = 100 μm. (**F**) Western blots of total proteins extracted from TC-1 tumors confirmed comparable levels of pimonidazole-protein adducts in the tumors grown in Cx37−/− and WT mice. Total protein stain (MemCode) was used to control for a comparable protein loading. The specificity of the pimonidazole staining was assessed using tumor samples of WT and Cx37−/− mice which had not been previously injected with the pimonidazole HCl (HypoxyprobeTM) and revealed no adducts’ signal (data not shown). Data are mean + SEM. WT (*n* = 8) and Cx37−/− (*n* = 7) mice (Student’s *t*-test).

Immunostaining of the s.c. TC-1 tumors for desmin and alpha-smooth-muscle actin (αSMA) showed that Cx37−/− and WT mice featured a similar coverage of the angiogenesis-derived capillaries by mural cells (Figure 4A–C). In addition, the i.v. injection of FITC-labeled tomato lectin revealed a comparable proportion of perfused vessels in the tumors grown in Cx37−/− and WT mice (Figure 4D). A combined immunostaining of pimonidazole and the EC marker VeCad was used to assess the distribution of hypoxic areas and of vessels, respectively. The percentage of hypoxic regions was similar in the TC-1 tumors grown in Cx37−/− and in WT mice (Figure 4E), a conclusion further supported by the levels of pimonidazole-protein adducts, as evaluated in Western blots (Figure 4F). The data show that the absence of Cx37 does not modify the structure and permeability of the vessels which newly formed within the tumors.

### 2.5. Loss of Cx37 Decreases Angiogenesis of TC-1 Tumors Induced within the Bladder

**Figure 5:**
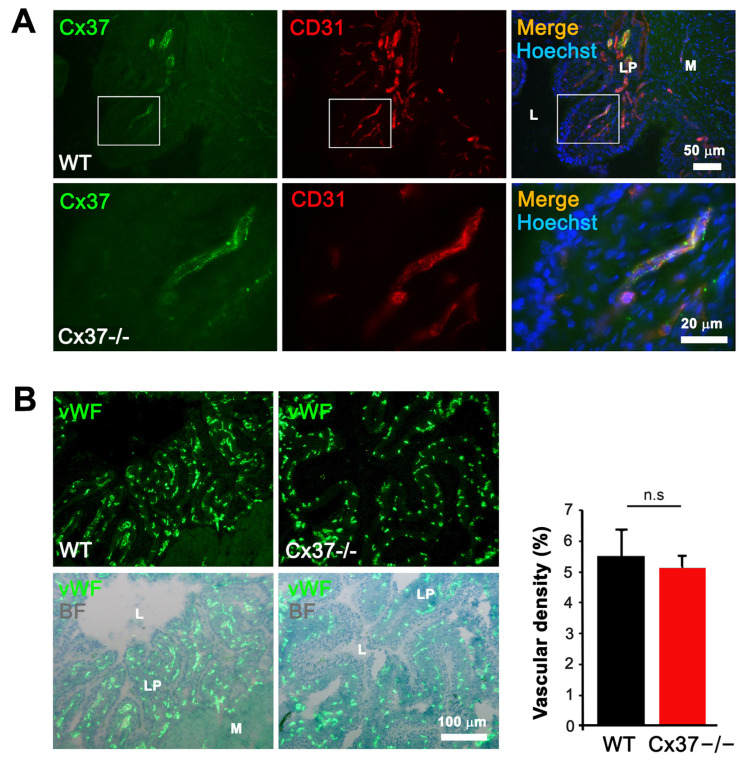

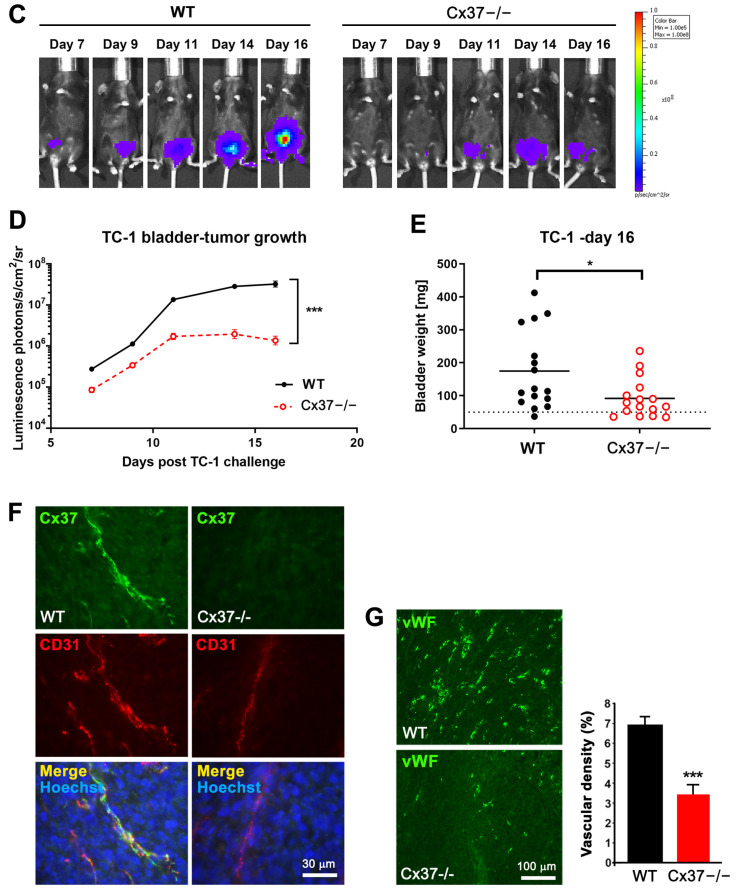
Loss of Cx37 decreases the vascularization and growth of TC-1 tumors established in the bladder. (**A**) Immunostaining showed Cx37 (green) in the CD31-positive EC (red) of control bladders. Representative images are shown at low (bar = 50 µm) and high magnification (bar = 20 µm) in the top and bottom row, respectively. Cell nuclei are seen after Hoechst staining (blue). (**B**) Immunostaining of native bladder sections for Von Willebrand factor (vWF, top panel), overlaid with Evan’s blue counterstain (bright field, BF, bottom panel), revealed many blood vessels, mainly localized within the lamina propria of the bladder (LP; L = lumen; M: muscular layers). Quantitative evaluation showed that the volume density of these vessels was similar in the bladders of WT and Cx37−/− mice. Data are mean + SEM values of 3 fields, photographed from 3 mice per group. (**C**) Representative bioluminescence imaging of TC-1-luc tumors growing in the bladder of a WT (left) and a Cx37−/− mouse (right). (**D**) The mean + SEM bioluminescence intensity (photons/sec/cm^2^/sr) of the TC-1 tumors growing in the bladder of WT mice (black line, *n* = 16) was significantly higher than that of the tumors growing in the bladder of Cx37−/− mice (red line, *n* = 16). *** *p* < 0.001 versus WT mice (Student’s *t*-test on the area under the tumor growth curve). (**E**) Sixteen days after the intravesical instillation of TC-1 cells, the combined weight of bladders and growing tumors was measured and found to be significantly higher in WT than Cx37−/− mice. Horizontal lines show mean values. The dotted line shows the mean weight of native mice bladders. * *p* < 0.05 versus WT mice (Student’s *t*-test). (**F**) Immunostaining showed the presence of Cx37 on CD31-positive EC of TC-1-luc tumors grown in the bladders of WT (left) but not of Cx37−/− mice (right). (**G**) Immunostaining for von Willebrand factor (vWF) revealed that the vessel density of TC-1-luc tumors was higher in Cx37−/− than WT mice. Data are mean + SEM of 5–7 areas from tumors of similar size, which developed after 16 days in 5 different mice per group. *** *p* < 0.001 versus WT mice (Student’s *t*-test).

Cx37 is detected in the bladder vessels of healthy WT mice (Figure 5A), which show a capillary network density similar to that of Cx37−/− mice (Figure 5A,B). Instillation of TC-1 cells induced, within one week, luminescence-detectable tumors in both WT and Cx37−/− mice. However, after 16 days, the tumors grown in the bladder of Cx37−/− mice were significantly smaller than those grown in WT mice, as judged by both luminescence measurements (Figure 5C,D) and the combined weight of bladders and tumors at sacrifice (Figure 5E), reflecting a reduced growth. Tumor capillaries expressing Cx37 were only identified in the CD31-positive EC of the tumors grown in WT mice (Figure 5F and Supplementary Figure S2). These tumors also exhibited a higher density of blood vessels than the tumors grown in Cx37−/− mice (Figure 5G). The data show that loss of Cx37 decreases the growth and angiogenesis in a tumor induced in the bladder, without affecting the vascularization of the host organ before tumor implantation.

### 2.6. Loss of Cx37 Decreases the Growth and Angiogenesis of B16-F10 Tumors

**Figure 6:**
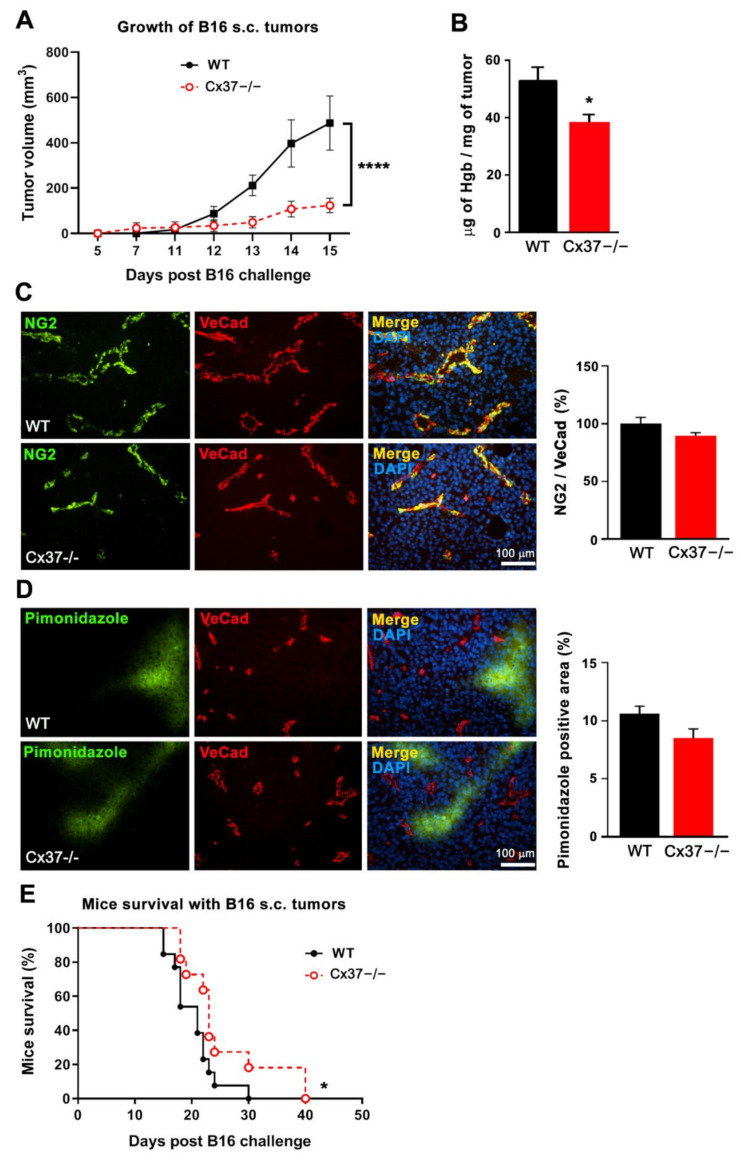
Loss of Connexin37 decreases the growth of B16-F10 tumors and extends the survival of the tumor-bearing mice. B16-F10 cells were s.c. injected to generate tumors in WT and Cx37−/− mice. (**A**) The volume of the tumors growing in Cx37−/− mice until day 15 was significantly smaller than that of the tumors growing in WT animals. Data are mean ± SEM of WT mice (*n* = 11, black line) and Cx37−/− mice (*n* = 13, dotted red line). **** *p*< 0.0001 versus WT mice (areas under the tumor growth curve were compared by Student’s *t*-test). (**B**) In another set of mice, which were sacrificed 14 days after the cell injection, the hemoglobin content of the tumors grown in Cx37−/− mice was lower than that evaluated in the WT controls. (**C**) Immunostaining showed a similar distribution of NG2 over the VeCad positive vessels of the B16-F10 tumors which grew in WT and Cx37−/− mice. (**D**) Immunostaining of pimonidazole (green) revealed hypoxic areas in tumor regions containing VeCad-positive vessels (red). Quantitative analysis revealed that the surface of these hypoxic areas was comparable in the tumors grown in Cx37−/− and WT mice. Data are mean + SEM of 8–15 fields from 7 mice per group (Student’s *t*-test). Bars = 100 μm. (**E**) The survival of the Cx37−/− mice that carried a B16 tumor was significantly increased compared to that of the cognate WT animals. Data are mean ± SEM of 11 WT mice (black line) and 13 Cx37−/− mice (dotted red line). * *p* < 0.05 versus WT mice (log-rank Mantel-Cox test).

Fifteen days after the s.c. implantation of B16-F10 cells, sizable tumors had grown in all WT mice (Figure 6A), whereas, at the same time point, Cx37−/− mice developed significantly smaller tumors (Figure 6A). B16-F10 tumors grown in Cx37−/− mice featured a lower hemoglobin content compared to tumors grown in WT mice (Figure 6B). However, the NG2-positive mural cells coverage of the vessels (Figure 6C) as well as the percentage of hypoxic regions were similar in the tumors grown in the two types of mice (Figure 6D). Under these conditions, the survival of Cx37−/− mice was significantly longer than that of WT animals (Figure 6E). The data show that lack of Cx37 decreases the angiogenesis and growth of B16-F10 tumors, thus prolonging the survival of the tumor-bearing mice, similarly to what was observed for TC-1 tumors.

### 2.7. A Peptide Targeting Cx40 Decreases Tumoral Growth in Cx37−/− Mice

**Figure 7:**
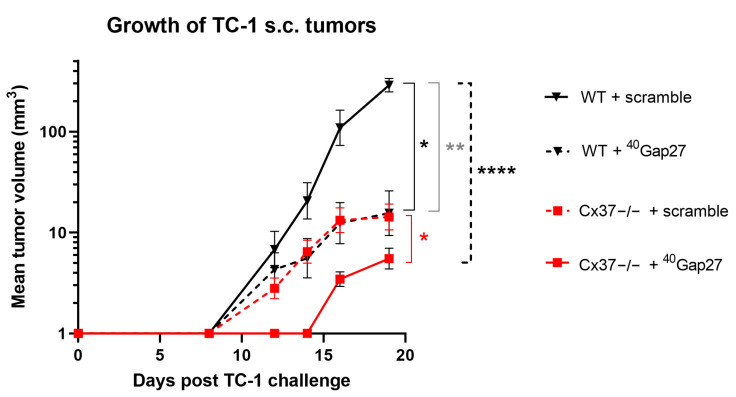
A peptide targeting Cx40 function decreases tumoral growth in Cx37−/− mice. One day after the s.c. injection of TC-1 cells, WT (*n* = 12, black lines) and Cx37−/− mice (*n* = 16, red lines) received a daily i.p. injection of either the ^40^Gap27 peptide, which specifically targets Cx40 channels, or its scrambled version, which served as control. The plot shows the mean ± SEM tumor volumes as a function of time. After 19 days, the growth of TC-1 tumors that received the scramble version of the peptide was significantly slower in Cx37−/− than in WT mice. In both groups of mice, the animals that received the ^40^Gap27 peptide developed smaller tumors than the animals which received its scrambled form. Significant differences in tumor growth, as judged by the area under the curve, were determined using a one-way ANOVA and a Sidak’s multiple comparisons post-test. Cx37−/− + scramble vs. WT + scramble (grey); Cx37−/− + scramble vs. Cx37−/− + 40Gap27 (red); WT + 40Gap27 vs. WT+ scramble (black); Cx37−/− + 40Gap27 vs. WT+ scramble (black dash line). * *p* < 0.05; ** *p* < 0.01; **** *p* < 0.0001.

Most primary EC co-express Cx37 and Cx40 and remain linked by these two proteins within tumors, implying that some of the effects attributed to the loss of Cx37 may be due to a change in Cx40. This possibility cannot be tested using mice null for both connexins, which rapidly die after birth [26]. To bypass this problem, we have treated Cx37-null mice with a specific peptide inhibiting Cx40 [28]. One day after the s.c. injection of TC-1 cells, WT and Cx37−/− mice received daily i.p. 100 μg of either ^40^Gap27, a peptide inhibiting Cx40 channels, or its scrambled version. As anticipated from the experiments summarized above, Cx37−/− mice that received the scrambled peptide featured a slower growth of tumors than the WT mice that received the same treatment (Figure 7). Within 2–3 weeks, all mice injected with the ^40^Gap27 peptide developed significantly smaller tumors than the animals that were injected with the scrambled control, but again the tumor growth was smaller in the Cx37−/− mice than in the WT mice (Figure 7). The data suggest that Cx40 inhibition adds to the effect of Cx37 loss in controlling the growth of TC-1 tumors.

## 3. Discussion

We investigated whether Cx37, a major connexin (Cx) of EC [18,22,30,35,42], is involved in the control of tumor angiogenesis. Using WT and Cx37-null mice [30,35,38], we show that loss of Cx37 induces in vivo a decreased angiogenesis of different experimental tumors, in spite of a similar structure and permeability of the newly formed vessels. This effect is associated with a reduced growth of tumoral cells, whether established subcutaneously or within the bladder, thereby significantly increasing the survival of the tumor-bearing animals.

Angiogenesis requires a fine-tuned coordination between the proliferation, migration and differentiation of both endothelial cells (EC), which form capillary-like tubes, and the recruitment of mural cells, which will form the pericyte and smooth muscle cell layers of the neovessels maturating towards a venous or an arterial phenotype [43]. The latter event of vascular maturation tubes is triggered by various factors secreted by EC, including PDGF isoforms, which act as chemo-attractants for mural cell precursors [10,11]. During tumor angiogenesis, the overexpression of pro-angiogenic factors results in the formation of irregular and tortuous vessels, which feature a discontinuous pericyte coverage, a high permeability [1,12] and a fenestrated basement membrane [13]. These features compromise the barrier integrity of the tumor vasculature and its perfusion, resulting in a sustained hypoxia, which may contribute to tumor maintenance and resistance to chemotherapy [14,15].

Compared to WT mice, the similar structure and permeability of the tumoral vessels, which developed in the absence of Cx37, contrast with the observations documenting that the loss of Cx37 transiently decreases the mural cells coverage of the capillaries which form during the physiological angiogenesis of the developing retina [30]. This alteration is probably accounted for by a change in the EC secretion of PDGF and/or Ang-2 [31,44]. Future studies should determine whether this difference is due to the age of the animals studied (neonatal in the retina studies, adult here), to the form of angiogenesis (physiological in the retina, tumoral here), to the duration of angiogenesis before the testing (a few days in the retina, a few weeks here) or to other experimental differences. Our study further reveals that the lower density of the newly formed vessels lacking Cx37, which we observed within the three tumoral models studied (subcutaneous TC-1 cells, intravesical TC-1-luc cells, subcutaneous B16-F10 cells), was not associated to obvious changes in the structural and functional maturation of the growing vessels, as reflected by a control coverage by mural cells, a control permeability and control areas of tumoral hypoxia, respectively. 

The obvious question is now to understand how Cx37 can regulate tumor growth and angiogenesis in response to various angiogenic factors and in different tumor models. It is unlikely that the Cx could achieve such effects by directly modulating tumor cells, inasmuch as B16-F10 [45] and TC-1 cells express Cx43, but not Cx40 and Cx37 (Supplementary Figure S3). At any rate, the effects of Cx37 and Cx40 are different. Thus, whereas the loss of Cx40 decreases the angiogenesis of tumor vessels featuring an increased pericyte coverage [28], here we show that the loss of Cx37 similarly decreases tumoral angiogenesis, without modifying the mural cell coverage of the newly formed capillaries. Since the expressions of Cx40 and Cx37 are co-regulated [16,21,29,30,46], one may question the mutual role of these two Cx. A previous study reported an up-regulation of Cx40 under conditions raising the expression of HIF-1a [47], still not in the context of angiogenesis. Our data show that tumor hypoxia was similar in the tumors grown in Cx37−/− and WT mice, indicating that HIF-1α is unlikely to account for the tumor differences observed in the two types of mice. We also reported that the role of Cx37 and Cx40 is not identical during the developmental angiogenesis of the retina [29,30] and now extend these observations in the context of tumoral angiogenesis. Specifically, we have shown that the pharmacological targeting of Cx40 reduced the in vivo growth of different tumor models [28]. Here, we show that the combined targeting of Cx37 and Cx40, after injection of a Cx40 inhibitory peptide into Cx37−/− mice, further reduced tumor growth, suggesting a collaborative effect of the two connexin isoforms. Thus, the use of connexin-targeting peptides provides a suitable way to test the effect of a combined knockdown of Cx37 and Cx40, given that mice lacking both proteins develop extensive hemorrhages which result in a rapid perinatal death [26]. 

In summary, both Cx37 and Cx40 significantly interfere with the in vivo growth of different tumors, by reducing their angiogenesis in a somewhat different manner. Proper identification of the respective roles of the different EC Cx will require the experimental testing of novel functional assays, providing conditions close to those prevailing in an in vivo tumor setting. Future investigations will indicate whether any such assay [48,49,50,51,52] can be designed to faithfully and simultaneously address these requirements. Further unravelling of the cell and molecular mechanisms which link Cx37 to tumoral angiogenesis and growth should provide novel insights to promote the development of innovative anti-tumoral treatments.

## 4. Materials and Methods

### 4.1. Cell Culture and Transfections

Primary human umbilical vein EC (HUVEC, C-12202, Promocell, Heidelberg, Germany) and primary human aortic EC (HAoEC, C-12271, Promocell, Heidelberg, Germany) were cultured in EGM-2 medium (CC-3162, Lonza, Basel, Switzerland). Cells were cultured at 37 °C under a 5% CO_2_ atmosphere and used until passage 10, by providing fresh medium every 2 days. Cx37 knockdown was performed by exposing the cells to the short interfering RNAs (si) siCx371 (s223720, Ambion-Life Technologies, Austin, TX, USA) or siCx374 (7095, Ambion-Life Technologies, Austin, TX, USA) and controlled use of siControl (siCtl, AllStars Negative Control siRNA, SI03650318, Qiagen, Hilden, Germany), as published [30]. To this end, HUVEC grown at 70% confluence were transfected overnight with 30 nM siRNAs, using lipofectamin RNAiMax (13778–075, Invitrogen, Waltham, MA, USA). After 48 h, the cells were collected, centrifuged and kept at −20 °C until analysis. HUVEC grown on glass coverslips were fixed for 5 min in −20 °C acetone, air-dried, rinsed in PBS, permeabilized for 1 h in PBS supplemented with 2% BSA and 0.1% Triton X-100 and immunostained for BrdU, as previously described [53,54]. BrdU positive nuclei were detected using ImageJ software [55] and normalized to the total number of DAPI-positive nuclei. TC-1 cells were also immunostained for Cx43 (3512, Cell Signaling Technology, Danvers, MA, USA), as reported [21,23,35]. No attempt was made to evaluate the EC proliferation in vivo, given that the BrdU staining could not easily differentiate the mitotic EC from the nearby, actively proliferating tumor cells. AllStars Negative Control siRNA was used as a control. SiRNA transfections were conducted using lipofectamin RNAiMax (Invitrogen, Waltham, MA, USA) at a final concentration of 30 nM, as published [29]. 

### 4.2. Connexin-Mimetic Peptides 

Various agents have been used as gap junction blockers, but their nonspecific effects have limited their use [56]. In contrast, the Cx inhibitory peptides are highly selective and specific [57]. These short synthetic peptides correspond to conserved amino acid sequences in the first (Gap 26) and/or second (Gap 27) extracellular loops of Cx37 and/or Cx40 and disrupt the function of the membrane channels made by these proteins [58,59,60,61]. Connexin-mimetic peptides were used to specifically inhibit Cxs. The sequences of the connexin-mimetic peptides ^40^Gap27 and ^37,43^Gap27 correspond to conserved amino acid sequences located in the second extracellular loops of Cx40 and Cx37, respectively [57,59,61,62]. The ^40^Gap27 sequence is Ser-Arg-Pro-Thr-Glu-Lys-Asn-Val-Phe-Ile-Val, which specifically targets the second extracellular loop region of Cx40 [40]. The cognate scrambled version is Thr-Phe-Glu-Pro-Val-Arg-Val-Ser-Ile-Asn-Lys. The ^37,43^Gap27 sequence is Ser-Arg-Pro-Thr-Glu-Lys-Thr-Ile-Phe-Ile-Ile, and the cognate scrambled sequence is Thr-Phe-Glu-Pro-Ile-Arg-Ile-Ser-Ile-Thr-Lys. Peptides were synthesized by the Protein and Peptide Chemistry Facility of the University of Lausanne, Switzerland, and reconstituted in sterile PBS.

### 4.3. Animals 

Experiments compared female Cx37−/− and WT littermates, maintained on a C57BL/6J background. The former mice were generated by breeding heterozygote Cx37 + /− males and females [21,30,34,35,38,63]. WT, heterozygous and knock-out animals were identified via PCR of genomic DNA, using the following primers: forward primer 5′-TCCCAAGGGCTTACATCCCA-3′ and reverse primer 5′-AGCAGCCTCTGTTCCACATAC-3′ to detect the Cx37 knock-out allele, and reverse primer 5′-AGCACGCTGACCACATAGGTA-3′ to detect the Cx37 wild-type allele [30,38]. Mice were housed and bred according to standard animal facility procedures. Mouse care and euthanasia procedures were approved by the institutional committees for animal experiments and by the veterinary office of Lausanne (Switzerland). Animal experimentation conformed to the Guide for the Care and Use of Laboratory Animals (NIH Publication Eighth Edition, 2011). 

### 4.4. Tumor Models 

Tumors were generated in WT and Cx37−/− mice by a s.c. injection of either 2 × 10^4^ TC-1 cells (26), which are mouse lung cells expressing the human papillomavirus oncogene [64], or 2.5 × 10^5^ B16-F10 cells, which are mouse melanoma cells [65]. Tumor growth was monitored every two days with a caliper, and tumor volume was given by length × width^2^ /2 [28]. Mice were sacrificed when tumor volumes were >1.5 cm^3^, as per veterinary guidelines. Orthotopic bladder tumors were generated by instilling 2.5 × 10^5^ TC-1-luc cells into the bladder, as previously described [28,66]. The growth of bladder tumors was monitored via bioluminescence 15 min after an i.p. injection of 150 mg/g b.w. D-luciferin (L8220, Promega, Madison, WI, USA,), using the Xenogen imaging system (Xenogen/IVIS Caliper Life Science, Hopkinton, MA, USA) of the cellular imaging facility at CIF/UNIL. Tumor bioluminescence was detected 7 days after the cell instillation and monitored until day 16 [67]. At this time, the animals were sacrificed, the size of the tumors was estimated by measuring the combined weight of bladders and their tumors, and all tissues were frozen into liquid nitrogen. In parallel experiments, WT and Cx37−/− mice were injected i.p. every day with 100 µg of either ^40^Gap27 peptide, which specifically targets Cx40 [40,41], or its scrambled version, starting one day after tumor cell implantation [28,29,39,40,41].

### 4.5. Matrigel Plug Assay

Plugs of 0.5 mL Growth Factor Reduced matrigel (356231, BD Biosciences, San Jose, CA, USA), supplemented with either 600 ng/mL recombinant murine FGF-2 (450-33, Peprotech, London, UK) and 3 U/mL heparin (Biochrom AG, L6510) or 200 ng/mL recombinant murine VEGF_165_ (450-32, Peprotech, London UK) and 10 U/mL Heparin, were s.c. injected into the flank of either WT or Cx37−/− mice. Matrigel plugs lacking growth factors were used as negative controls. WT mice were also injected with Growth Factor Reduced matrigel supplemented with 200 ng/mL VEGF, 10 U/mL heparin and 300 µM of either the ^37,43^Gap27 peptide or its scrambled version. The plugs were removed 1 week later for evaluation of vascularization, as published [28]. Specifically, the macroscopic observations of all implanted plugs showed the presence of blood vessels, and the color difference of the plugs implanted in control mice and Cx37−/− mice paralleled a different hemoglobin content, providing an indirect, still reliable and commonly used indicator of angiogenesis [28]. 

### 4.6. Immunostaining

At this stage, 8–10 μm-thick cryosections of bladder and s.c. tumors were exposed to blocking buffer (PBS, 2% BSA, 0.3% Triton X-100) for 1 h at room temperature and then incubated overnight at 4 °C in PBS 2% BSA with one of the following antibodies against: CD31 (553371, BD Pharmingen, San Diego, CA, USA), diluted 1:100; Cx37 (Cx37-A, Alpha Diagnostic International, San Antonio, TX, USA), diluted 1:100; Von Willebrand Factor (VWF, A0082, Dako, Wiesentheid, Germany), diluted 1:500; alpha-smooth muscle actin (αSMA, ab5694, Abcam, Cambridge, UK), diluted 1:200; VE-cadherin (555289, VeCad, BD Pharmingen, San Diego, CA, USA), diluted 1:100; desmin (5335, Cell Signaling, Danvers, MA, USA), diluted 1:500; chondroitin sulfate proteoglycan NG2 (AB 5320, Millipore, Volketswil, Switzerland,), diluted 1:500; cleaved caspase 3 (9661, Cell Signaling Technology, Danvers, MA, USA), diluted 1:100. Slides were then incubated for 1 h at room temperature with either Alexa-Fluor-594- or -488-coupled secondary antibodies (Life Technologies, Carlsbad, CA, USA), diluted 1:500, covered with PBS containing 50% glycerol and 0.4 μg/mL DAPI and observed using fluorescence microscopy (Nikon90i). The vascular density in tumors and native bladders (expressed as the percentage of VWF-positive areas per microscopic field) was quantified using the ImageJ software, as previously published [28].

### 4.7. Hemoglobin Content

Hemoglobin content was determined using the Drabkin’s reagent kit (Sigma Chemie, Buchs, Switzerland). Briefly, 100 mg tumor powder or excised matrigel plugs were homogenized in 0.5 mL 0.1% Brij-35 lysis buffer and centrifuged for 5 min at 10,000× *g*. Then, 50 μL of the supernatant was mixed with 450 μL Drabkin’s reagent and absorbance read at 540 nm. The concentration of hemoglobin was calculated from a cyan-methemoglobin standard curve [28]. 

### 4.8. Tumor Perfusion and Hypoxia Assays

Mice were i.v. injected with 100 mg FITC-tomato lectin (FL-1171, Vector, Burlingame, CA, USA), diluted in PBS. Thirty minutes later, mice were sacrificed, perfused with 4% paraformaldehyde through the left heart ventricle and the tumor sampled. Tumors were fixed in 4% paraformaldehyde overnight at 4 °C and then incubated in 30% sucrose overnight at 4 °C. The tissues were then embedded in OCT compound, cut at 20 μm thickness and kept frozen at −80 °C. CD31 was immunostained as described above. Cell nuclei were counterstained with DAPI and sections observed via fluorescence microscopy. For assessing hypoxia, 60 mg/Kg b.w. pimonidazole HCl (Hypoxyprobe^TM^) were i.v. injected into the mice, 60 min before sacrifice [68]. Tumors were then excised and sectioned. Sections were immunostained using the anti-pimonidazole, FITC-conjugated IgG1 antibody of the HypoxyprobeTM Plus Kit (hpi, Hypoxyprobe, Inc, Bulington, MA, USA) and VeCad, as described above. The sections were covered with PBS containing 50% glycerol and 0.4 μg/mL DAPI and observed by fluorescence microscopy (Nikon90i). Pimonidazole positive areas were quantified using ImageJ software. The specificity of the pimonidazole staining was assessed using tumor samples of WT and Cx37−/− mice which had not been previously injected with the pimonidazole HCl and in which no fluorescence signal would be expected. This was confirmed in Western blots of tumor protein extracts, which showed abundant pimonidazole positive adducts in samples of tumors from both WT and Cx37−/− mice injected with pimonidazole HCl but no signal in samples of mice that had not received the hypoxia probe (data not shown).

### 4.9. Protein Analysis

First, 100 mg powder of TC-1, B16-F10 tumors or confluent HUVEC cultures collected from a 12-well plate were homogenized via sonication in SDS Lysis Buffer (62.5 mM Tris-EDTA, pH 6.8, 5% SDS). Protein content was measured using a detergent-compatible protein assay kit (Bio-Rad Laboratories, Reinach BL, Switzerland). Then, 25 μg proteins were loaded on a 10% polyacrylamide gel, electrophoresed and transferred onto PVDF membrane (Immobilon-P; Millipore, Volketswil, Switzerland). Membranes were incubated for 1 h in TBS containing 5% milk and 0.1% Tween20 (blocking buffer). Saturated membranes were incubated overnight at 4 °C with the following antibodies against: VE-cadherin (sc-6458, Santa Cruz Biotechnology, Dallas, TX, USA), diluted 1:500; alphaSMA (ab5694, Abcam, Cambridge, UK), diluted 1:1000; desmin (ab15200, Abcam, Cambridge, UK), diluted 1:2000; Cx37 [30], diluted 1:2500; Cx40 (36-4900, diluted 1:1000, Life Technologies, Carlsbad, CA, USA); Cx43 (3512S, 1:1000, Cell Signaling,); pimonidazole (Hypoxyprobe^TM^ Plus Kit (hpi Hypoxyprobe, Inc., Bulington, MA, USA), diluted 1:1000; GAPDH (ab22555, Abcam, Cambridge, UK), diluted 1:3000; alpha-tubulin (T6074, Sigma-Aldrich, Buchs, Switzerland), diluted 1:30,000. Membranes were then incubated at room temperature for 1 h with a relevant secondary antibody conjugated to horseradish peroxidase (FlukaChemie, Buchs, Switzerland) diluted 1:20,000. Bands were developed using enhanced chemiluminescence (Immunobilon Western Chemiluminescent HRP substrate, Millipore, Volketswil, Switzerland) and visualized using a supercooled CCD camera (Chemidoc XRS, Bio-Rad Laboratories, Cressier, Switzerland). Densitometric analysis was performed using ImageLab Software (3.0.1 Bio-Rad Laboratories, Cressier, Switzerland). For hypoxia analysis, the levels of pimonidazole protein adducts were normalized to the amount of total proteins stained with the Pierce reversible protein Stain Kit (MemCode, 24585, Thermo Scientific, Reinach, Switzerland). The specificity of the pimonidazole staining was assessed using tumor samples of WT and Cx37−/− mice which had not been previously injected with pimonidazole HCl (HypoxyprobeTM, Bulington, MA, USA).

### 4.10. Statistics

Statistical analyses were performed using the Prism 8.0 software for Windows (GraphPad). Comparisons of group means were performed using either a *t*-test, log rank test, one-way ANOVA or two-way ANOVA, followed by Tukey’s or Sidak post hoc tests, whichever applicable. A *p*-value < 0.05 was taken to reflect a significant difference.

## 5. Conclusions

Our data document that Cx40 and Cx37 decrease tumoral angiogenesis, leading to a decrease in tumor growth and to longer survival of the tumor-bearing animals. These findings open the challenging perspective that the generation of tools to specifically target Cx40 and Cx37 may become useful for reducing angiogenesis and helping in the treatment of cancer patients.

## Data Availability

Data are presented in figures and Supplementary Figures.

## Supplementary Materials

The following supporting information can be downloaded at: https://www.mdpi.com/article/10.3390/ijms23062930/s1.
