# Correction: Sathiyanadan et al. Targeting Endothelial Connexin37 Reduces Angiogenesis and Decreases Tumor Growth. *Int. J. Mol. Sci.* 2022, *23*, 2930

**DOI:** 10.3390/ijms27052494

**Published:** 2026-03-09

**Authors:** Karthik Sathiyanadan, Florian Alonso, Sonia Domingos-Pereira, Tania Santoro, Lauriane Hamard, Valérie Cesson, Paolo Meda, Denise Nardelli-Haefliger, Jacques-Antoine Haefliger

**Affiliations:** 1 Department of Urology, Lausanne University Hospital, 1011 Lausanne, Switzerland; karthik.sathiyanadan@gmail.com (K.S.); sonia.domingos-pereira@chuv.ch (S.D.-P.);; 2 Laboratory for the Bioengineering of Tissues (BioTis-INSERM U1026), Université de Bordeaux, 33607 Bordeaux, France; f.alonso@iecb.u-bordeaux.fr; 3 Department of Medicine, Lausanne University Hospital, 1011 Lausanne, Switzerland; tania.santoro@chuv.ch (T.S.);; 4 Department of Cell Physiology and Metabolism, Medical Center, University of Geneva, 1206 Geneva, Switzerland

In the original publication [1], there was a mistake in Figure 5G as published. In this figure, we inadvertently used the same representative image that had previously been published in Figure 2B of our article [2]. We sincerely apologize for this unintentional error. The corrected Figure 5 and Figure caption appears below. The authors state that the scientific conclusions are unaffected. This correction was approved by the Academic Editor. The original publication has also been updated.

## Figures and Tables

**Figure 5:**
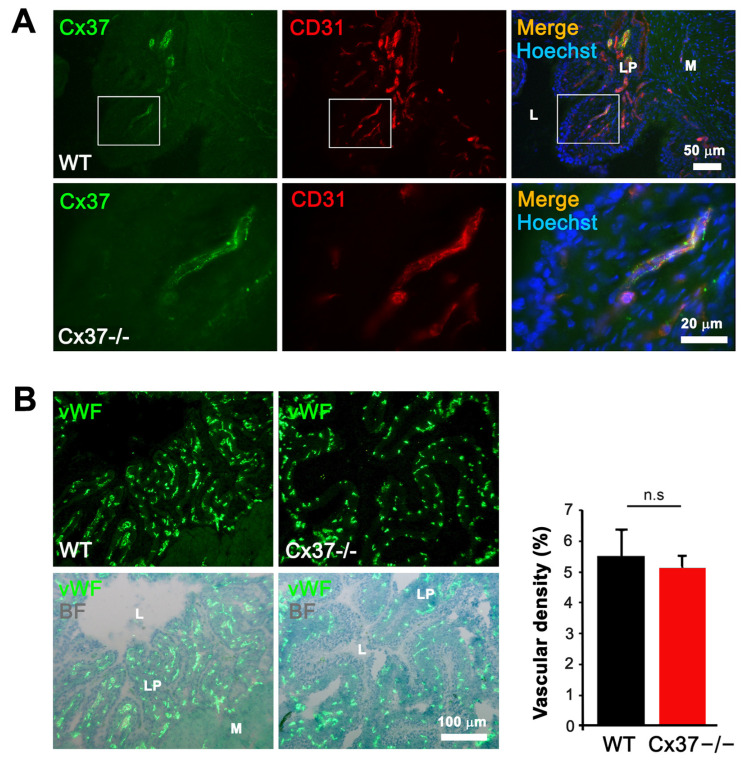

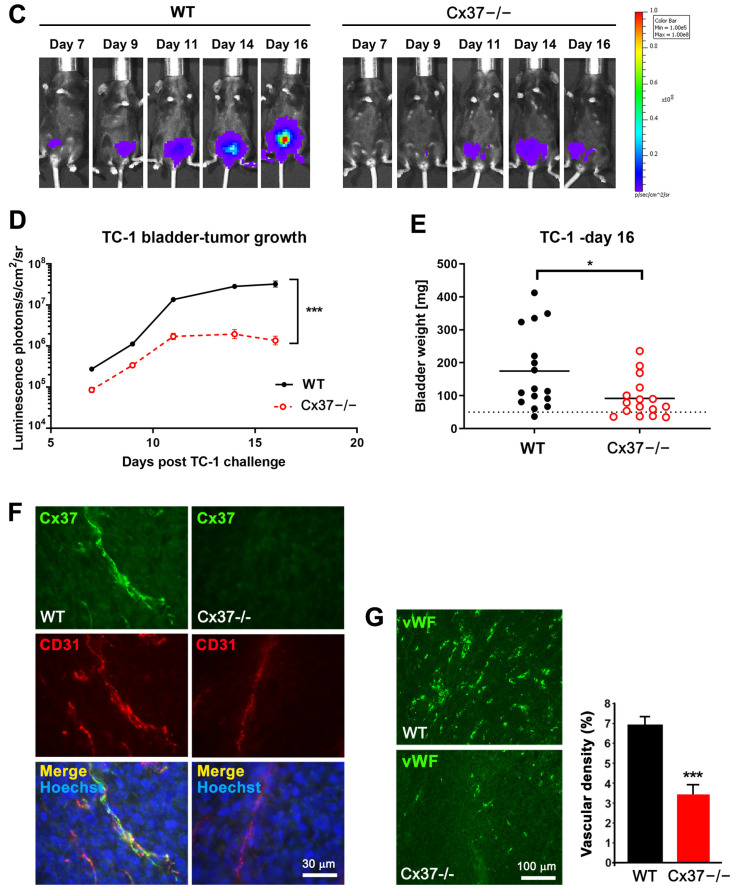
Loss of Cx37 decreases the vascularization and growth of TC-1 tumors established in the bladder. (**A**) Immunostaining showed Cx37 (green) in the CD31-positive EC (red) of control bladders. Representative images are shown at low (bar = 50 µm) and high magnification (bar = 20 µm) in the top and bottom row, respectively. Cell nuclei are seen after Hoechst staining (blue). (**B**) Immunostaining of native bladder sections for Von Willebrand factor (vWF, top panel), overlaid with Evan’s blue counterstain (bright field, BF, bottom panel), revealed many blood vessels, mainly localized within the lamina propria of the bladder (LP; L = lumen; M: muscular layers). Quantitative evaluation showed that the volume density of these vessels was similar in the bladders of WT and Cx37−/− mice. Data are mean + SEM values of 3 fields, photographed from 3 mice per group. (**C**) Representative bioluminescence imaging of TC-1-luc tumors growing in the bladder of a WT (left) and a Cx37−/− mouse (right). (**D**) The mean + SEM bioluminescence intensity (photons/sec/cm^2^/sr) of the TC-1 tumors growing in the bladder of WT mice (black line, *n* = 16) was significantly higher than that of the tumors growing in the bladder of Cx37−/− mice (red line, *n* = 16). *** *p* < 0.001 versus WT mice (Student’s *t*-test on the area under the tumor growth curve). (**E**) Sixteen days after the intravesical instillation of TC-1 cells, the combined weight of bladders and growing tumors was measured and found to be significantly higher in WT than Cx37−/− mice. Horizontal lines show mean values. The dotted line shows the mean weight of native mice bladders. * *p* < 0.05 versus WT mice (Student’s *t*-test). (**F**) Immunostaining showed the presence of Cx37 on CD31-positive EC of TC-1-luc tumors grown in the bladders of WT (left) but not of Cx37−/− mice (right). (**G**) Immunostaining for von Willebrand factor (vWF) revealed that the vessel density of TC-1-luc tumors was higher in Cx37−/− than WT mice. Data are mean + SEM of 5–7 areas from tumors of similar size, which developed after 16 days in 5 different mice per group. *** *p* < 0.001 versus WT mice (Student’s *t*-test).
