# Supplementary material for: Targeting Endothelial Connexin37 Reduces Angiogenesis and Decreases Tumor Growth
Source: Int J Mol Sci. 2022 Mar 8;23(6):2930. doi: 10.3390/ijms23062930 (PMC8948817; doi:10.3390/ijms23062930)
Supplement: Supplementary file 1 [file ijms-23-02930-s001.zip › ijms-1603257-supplementary.pdf]

Supplementary Materials:

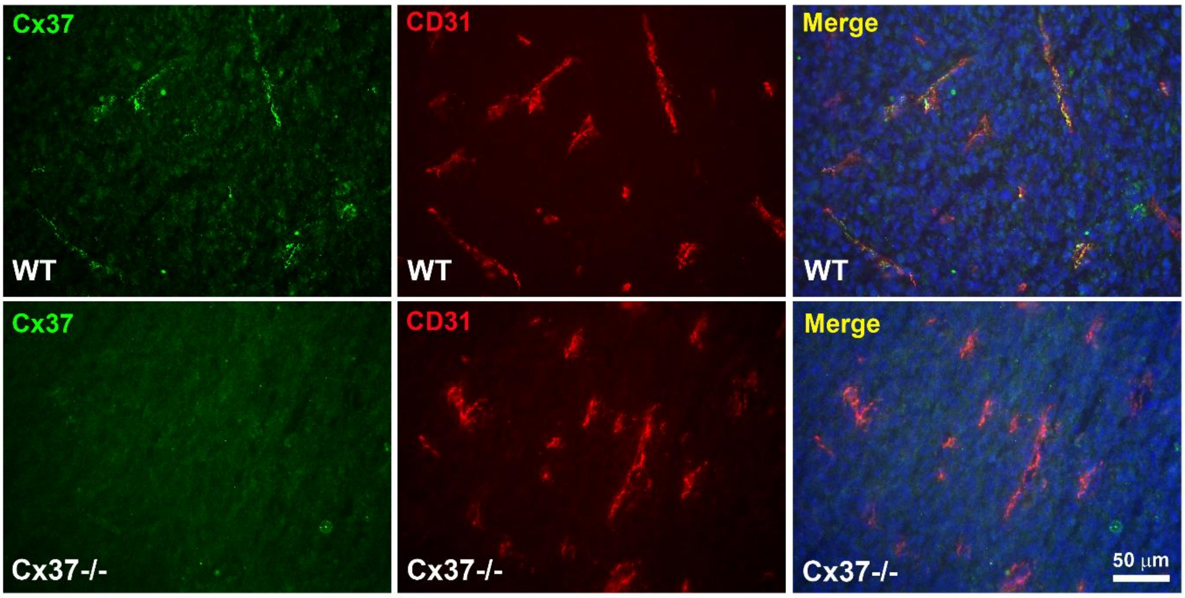

Supplementary Figure S1: Cx37 colocalizes with the EC marker CD31 in subcutaneous TC1 tumors. Double-staining of the specific EC marker CD31 and Cx37 was consistent in the EC of subcutaneous TC-1 tumors grown in WT and Cx37-/- mice. Cx37 was not detected between the TC1-luc tumor cells, nor in Cx37-/- mice. Bar, 50 μm.

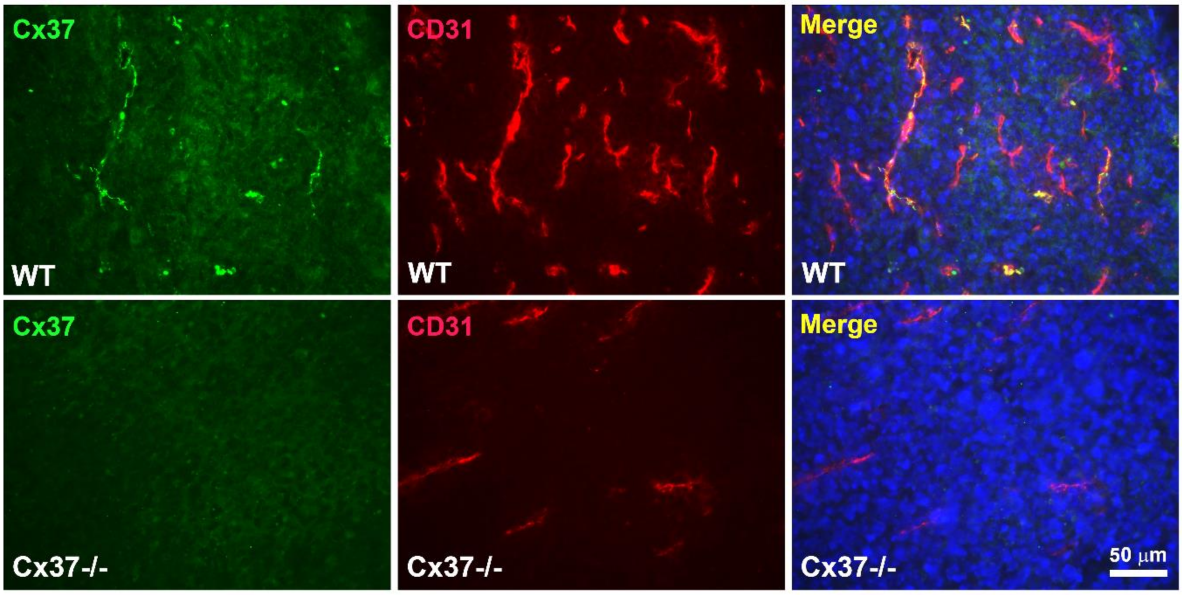

Supplementary Figure S2: Cx37 colocalizes with the EC marker CD31 in bladder TC1 tumors. Double-staining of the specific EC marker CD31 and Cx37 was consistent in the EC of TC-1 tumors grown in the bladders of WT and Cx37-/- mice. Cx37 was not detected between the TC1-luc tumor cells, nor in Cx37-/- mice. Bar, 50 μm.

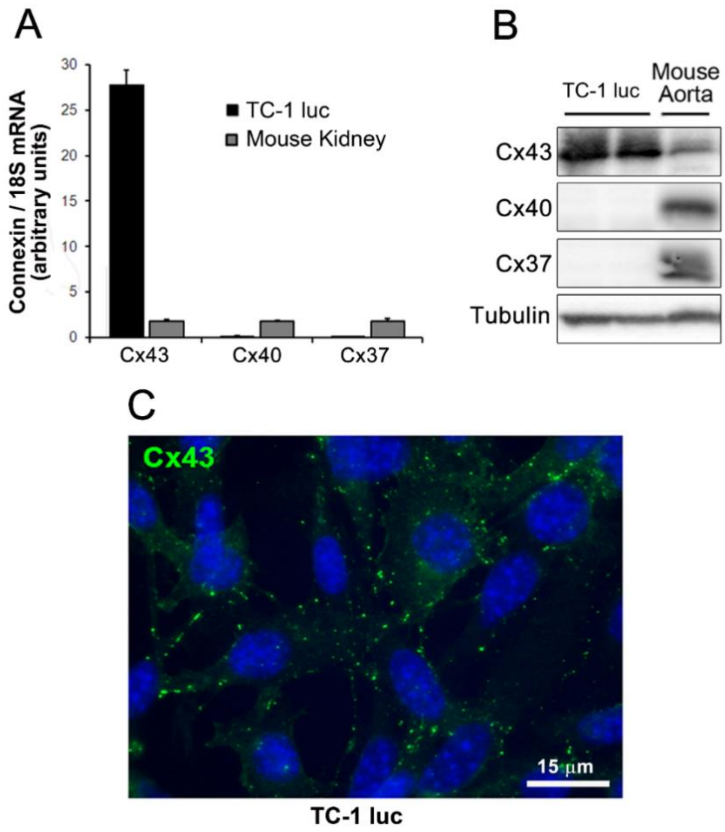

Supplementary Figure S3: Connexin expression in cultured TC-1 cells**. A**. RT-PCR revealed the transcripts coding for Cx43, Cx40 and Cx37 in mouse kidney, which served as positive control. Using the same probes, cultured TC-1 cells were found to express only the Cx43 transcripts. **B**. Western blots also identified Cx37, Cx40 and Cx43 in mouse aortas, which served as positive controls, but only detected the Cx43 protein in cultured TC-1 cells. **C**. immunofluorescence showed the punctate distribution of the later connexin isoform at contacting membranes of TC-1 cells.
